# Nutritional status and systemic inflammation in COPD: prognostic value of the advanced lung cancer inflammation index

**DOI:** 10.3389/fnut.2025.1550490

**Published:** 2025-04-28

**Authors:** Jun Yao, Peng Wu, Zhishu Li, Lingyan Zhao, Ziqiao Fu, Ping Shi, Xiaomin Xiong, Xuping Chen, Bin Yu, Yan He, Tong Feng, Jia Zeng, Ran Duan

**Affiliations:** ^1^Department of Respiratory and Critical Care, Guangyuan Central Hospital, Sichuan, China; ^2^Department of Respiratory and Critical Care Medicine, Deyang People’s Hospital, Affiliated Hospital of Chengdu College of Medicine, Deyang, China; ^3^School of Clinical Medicine, Chengdu Medical College, Chengdu, Xindu, China; ^4^Department of Oncology, The First Affiliated Hospital of Chengdu Medical College, Chengdu, China

**Keywords:** chronic obstructive pulmonary disease, advanced lung cancer inflammation index, all-cause mortality, cardiovascular mortality, National Health and Nutrition Examination Survey

## Abstract

**Background:**

Chronic Obstructive Pulmonary Disease (COPD) is a leading cause of global mortality, with systemic inflammation and malnutrition playing pivotal roles in its progression and outcomes. The Advanced Lung Cancer Inflammation Index (ALI), which integrates nutritional status and systemic inflammation, may offer potential prognostic value in COPD management.

**Objectives:**

This study aimed to evaluate the relationship between ALI and mortality outcomes in COPD patients, with a specific focus on the interplay between nutrition, inflammation, and their non-linear associations with all-cause and cardiovascular mortality.

**Methods:**

Data were derived from the NHANES (1999–2018) cohort, comprising 47,880 participants, including 1,960 COPD patients. ALI was calculated using body mass index (BMI), serum albumin levels, and the neutrophil-to-lymphocyte ratio (NLR). Survival analyses, including Kaplan–Meier curves, Cox proportional hazards models, and restricted cubic splines, were used to assess the association between ALI and mortality outcomes, exploring non-linear trends and thresholds.

**Results:**

Higher ALI levels were significantly associated with reduced risks of all-cause and cardiovascular mortality in COPD patients. Protective effects plateaued at ALI thresholds (88.32 for all-cause mortality and 89.73 for cardiovascular mortality), with mortality risks reversing at excessively high levels for cardiovascular mortality.

**Conclusion:**

ALI, as a composite marker of nutritional status and systemic inflammation, is a valuable prognostic tool for COPD patients. Its non-linear relationship with mortality underscores the need to optimize nutritional and inflammatory management strategies. These findings emphasize the critical importance of addressing malnutrition and systemic inflammation to improve COPD outcomes. Future research should validate these findings and investigate tailored nutritional interventions and anti-inflammatory treatments.

## Introduction

Chronic Obstructive Pulmonary Disease (COPD) is a prevalent and debilitating condition characterized by persistent respiratory symptoms and airflow limitation, with a substantial impact on global health. According to the World Health Organization, more than 330 million individuals worldwide are affected by COPD, which is projected to become the third leading cause of mortality by 2030 (1). Identifying modifiable factors that can improve long-term outcomes for COPD patients is crucial to alleviating the disease burden and reducing premature deaths.

Inflammation is a key driver of COPD progression, initiated by exposure to harmful particles such as cigarette smoke, air pollution, and occupational irritants. These stimuli activate epithelial cells and alveolar macrophages in the lungs, leading to the release of pro-inflammatory cytokines, including tumor necrosis factor-alpha (TNF-*α*), interleukin-6 (IL-6), and interleukin-8 (IL-8) (2). These mediators recruit neutrophils, macrophages, and CD8+ T cells to the airways, resulting in tissue damage, airway remodeling, and impaired mucociliary clearance. However, focusing solely on inflammation without considering the patient’s nutritional status may provide an incomplete understanding of its impact on disease progression and outcomes. Serum albumin, a critical marker of nutritional status and systemic inflammation, often declines in COPD patients due to chronic inflammation, oxidative stress, and reduced hepatic synthesis. A recent meta-analysis of 26 studies found that COPD patients have significantly lower serum albumin levels compared to individuals without COPD (3). Additionally, individuals with low body mass index (BMI) frequently experience muscle wasting and reduced respiratory muscle strength, which exacerbate dyspnea and functional limitations. Chronic exposure to smoke can accelerate the aging process, resulting in reduced body weight and pulmonary aging (4). This acceleration may explain the observed strong correlation between low BMI and increased mortality in COPD patients. Furthermore, analyses from the ECLIPSE cohort, which includes patients with GOLD stage 2–4 COPD, suggest that the presence of cachexia is a significant predictor of mortality in COPD (5). Integrating inflammatory and nutritional markers offers a more comprehensive approach to predicting outcomes in COPD patients.

The Advanced Lung Cancer Inflammation Index (ALI), which combines markers of systemic inflammation and nutritional status, has shown promise in predicting outcomes in various conditions, including cancer and cardiovascular diseases (6, 7). Its proven prognostic value suggests it may also serve as a useful tool for assessing long-term prognosis in COPD patients, although its utility in this population remains understudied.

This study aims to provide new insights into the determinants of long-term prognosis in COPD patients by evaluating the role of ALI. By identifying potential intervention strategies, the research seeks to improve quality of life and survival outcomes for individuals living with COPD.

## Methods

This study utilized data from the National Health and Nutrition Examination Survey (NHANES), a nationally representative, ongoing cross-sectional survey conducted by the National Center for Health Statistics (NCHS). NHANES combines structured interviews, physical examinations, and laboratory tests to collect comprehensive health and nutritional data from the U.S. population. A multistage, stratified probability sampling method ensures its representativeness.

### Study population

**Figure 1:**
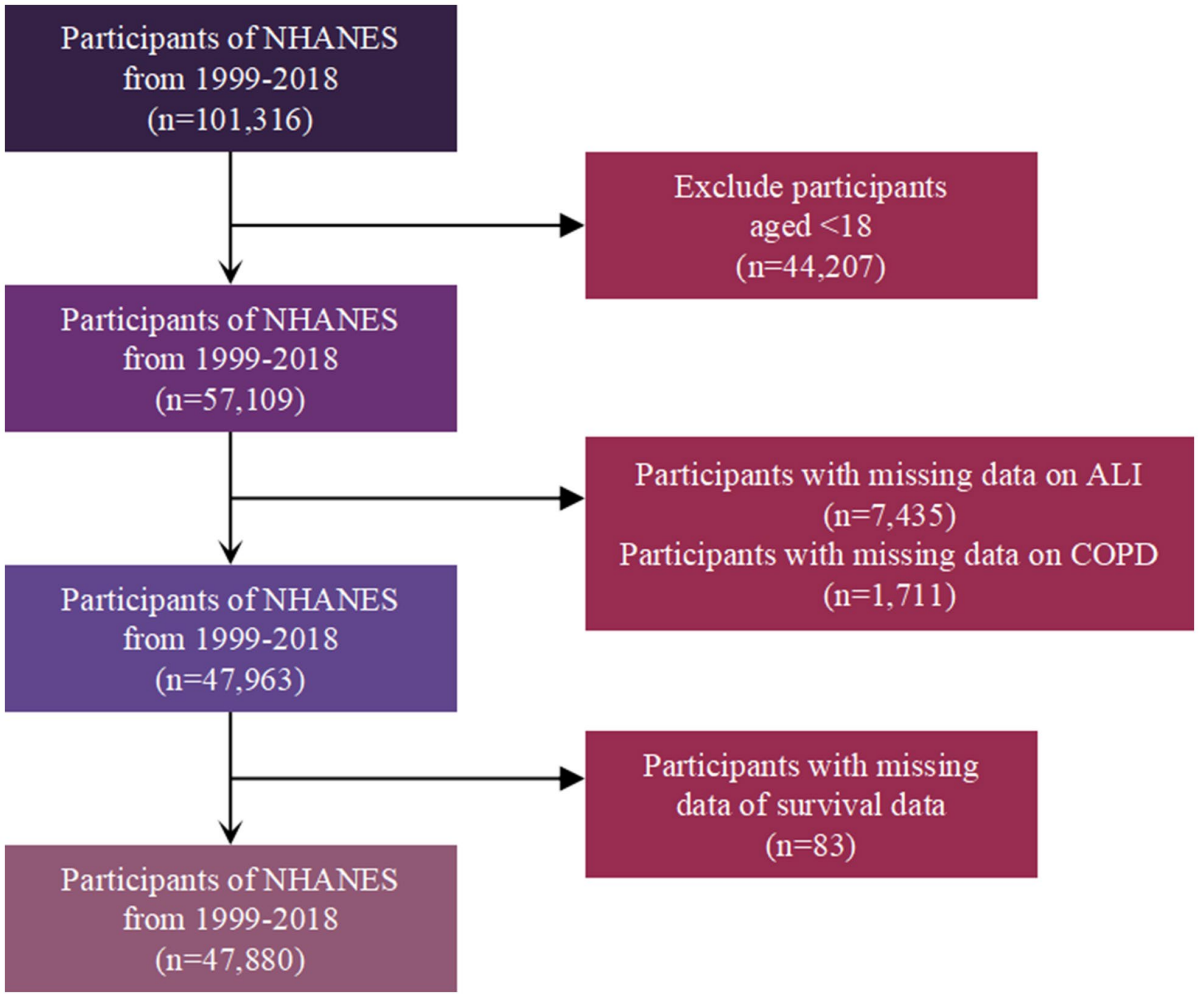
Flow chart of study participants.

The study initially included 101,316 participants from the NHANES 1999–2018 cohort. After excluding 44,207 individuals under 18 years old, 57,109 participants remained. Subsequently, 7,435 participants with missing data on ALI and 1,711 participants with missing data on COPD status were removed, leaving 47,963 participants. Finally, 83 participants with missing survival data were excluded, resulting in a final study population of 47,880 participants for analysis (Figure 1).

### COPD diagnosis

COPD was defined using a combination of spirometry results, self-reported medical history, medication usage, and specific risk factors. Participants were classified as having COPD if they met any of the following criteria: (1) a spirometry-based diagnosis, defined as a post-bronchodilator forced expiratory volume in 1 s (FEV₁) to forced vital capacity (FVC) ratio of less than 0.7 (FEV₁/FVC < 0.7), with spirometry data limited to tests graded as “A” or “B” for reliability; (2) a self-reported diagnosis, indicated by answering “yes” to either “Have you ever been told by a doctor or other health professional that you had emphysema?” (mcq160g); or (3) a medication-based diagnosis, defined as the current use of COPD-specific medications, including selective phosphodiesterase-4 inhibitors, mast cell stabilizers, leukotriene modifiers, or inhaled corticosteroids, combined with being aged 40 years or older and having a history of smoking or reported chronic bronchitis symptoms.

### Pulmonary function testing and assessment of chronic pulmonary symptoms

Pulmonary function was assessed through spirometry performed in the NHANES Mobile Examination Center (MEC) by trained technicians following standardized protocols. Both pre-bronchodilator and post-bronchodilator measurements of FEV₁ and FVC were recorded, with participants inhaling a bronchodilator medication (albuterol) prior to the post-bronchodilator test. The quality and validity of the spirometry data were evaluated according to American Thoracic Society (ATS) guidelines, and only tests graded as “A” (excellent quality) or “B” (good quality) were included in the analysis.

Chronic pulmonary symptoms were assessed through a standardized questionnaire administered during the NHANES interview. Participants were asked the following questions regarding respiratory symptoms:

Frequent cough: “Do you cough on most days for three consecutive months or more during the year?”

Frequent phlegm: “Do you bring up phlegm (thick mucus) on most days for three consecutive months or more during the year?”

Past-year wheeze: “Have you had wheezing or whistling in your chest at any time in the past 12 months?”

### Assessment of ALI

The ALI was assessed as a composite measure reflecting nutritional status and systemic inflammation. It comprised three components: BMI, serum albumin, and the neutrophil-to-lymphocyte ratio (NLR). The index was calculated using the formula: BMI (kg/m^2^) × albumin level (g/dL) ÷ NLR, where NLR was determined by dividing neutrophil counts by lymphocyte counts. BMI was derived from measured height and weight, serum albumin levels were obtained from blood samples, and NLR was calculated from complete blood count data. Higher ALI values indicated better nutritional reserves and lower systemic inflammation. For analysis, ALI was categorized into four groups: Minimal (<44.43), Low (44.43–60.92), Intermediate (60.92–81.9), and High (>81.9).

### Mortality outcomes

Mortality outcomes, including all-cause mortality and Cardiovascular disease (CVD)-specific mortality, were determined by linking NHANES participant data to the National Death Index (NDI) records through December 31, 2019. The NDI provides verified and detailed mortality data, including date and cause of death, using standardized codes based on the International Classification of Diseases, 10th Revision (ICD-10). All-cause mortality was defined as death from any cause, while CVD-specific mortality was identified using ICD-10 codes I00–I99. Mortality status and follow-up duration were calculated from the date of NHANES examination to the date of death or the end of the follow-up period.

### Covariates definitions

Self-reported sociodemographic characteristics included age (in years), gender (male or female), and race/ethnicity (Mexican American, Non-Hispanic Black, Non-Hispanic White, Other Hispanic, or Other Race, including multiracial individuals). Educational attainment was categorized as less than high school, high school or equivalent, or college and above. The family income-to-poverty ratio (PIR) was used as a measure of socioeconomic status and analyzed as a continuous variable.

Lifestyle factors included smoking status (never, former, or current smoker) and alcohol consumption, categorized as never, former, mild, moderate, or heavy drinker. Physical activity levels were assessed using Metabolic Equivalent of Task (MET) scores, recorded in minutes per week, and dietary quality was evaluated using the Healthy Eating Index-2015 (HEI-2015), where higher scores indicated better adherence to dietary guidelines.

Health-related conditions were determined based on self-reported diagnoses, laboratory tests, or clinical measurements. Diabetes mellitus (DM) was defined by a self-reported diagnosis, current use of insulin or antidiabetic medications, or laboratory findings, including fasting plasma glucose levels ≥7.0 mmol/L (126 mg/dL), glycated hemoglobin (HbA1c) ≥6.5%, or a 2-h plasma glucose level ≥ 11.1 mmol/L (200 mg/dL) from an oral glucose tolerance test. Hypertension was defined as a self-reported diagnosis, current use of antihypertensive medications, or measured systolic blood pressure ≥ 140 mmHg or diastolic blood pressure ≥ 90 mmHg, based on the average of three standardized blood pressure measurements taken during the NHANES physical examination.

Cancer status was categorized as yes or no based on participants’ history of cancer diagnosis. CVD was defined as self-reported diagnosis of any of the following: coronary heart disease, angina, stroke, myocardial infarction, or heart failure.

### Statistical analysis

Survey design and weights recommended by NHANES were applied to ensure the national representativeness of the results. Continuous variables were presented as means ± standard error. Categorical variables were reported as counts (percentages). Baseline characteristics among groups were compared using an analysis of variance test for continuous variables and the chi-square (χ^2^) test for categorical variables.

The association between ALI with all-cause and CVD mortality was evaluated using Kaplan–Meier survival analysis and Cox proportional hazards regression models. Three models were constructed for the Cox regression analysis: Model 1: Unadjusted crude analysis. Model 2: Adjusted for basic demographic and lifestyle factors (e.g., age, sex, race/ethnicity, marital status, family income, and educational attainment). Model 3: Fully adjusted for all covariates, including comorbidities, e.g., diabetes, hypertension, cancer, CVD, smoking status, alcohol consumption, physical activity (MET scores), and HEI-2015 scores. Because dividing ALI by 10 makes the odds ratio (OR) or hazard ratio (HR) more clinically interpretable, we divided each participant’s ALI level by 10 and included them as continuous variables in the multivariate Cox regression analysis.

Subgroup analyses were performed to explore the relationship between ALI and CVD mortality in various populations, such as those stratified by age, gender, smoking status, and comorbidities. Restricted cubic splines were used to investigate potential non-linear relationships between ALI with all-cause and CVD mortality.

Additionally, a propensity score matching (PSM) analysis was conducted to mitigate confounding effects. COPD patients were matched with non-COPD individuals using the nearest neighbor method. The balance of baseline covariates after matching was assessed using standardized mean differences, and Cox regression analysis was repeated in the matched cohort to confirm the robustness of the findings. To reduce the potential for reverse causation, sensitivity analyses excluded participants who died within the first 2 years of follow-up.

A two-sided *p*-value <0.05 was considered statistically significant for all analyses. All statistical analyses were conducted using R software (version 4.4.1; R Foundation for Statistical Computing, Vienna, Austria).

## Results

In this study, we aimed to explore the relationship between the ALI and mortality outcomes in COPD patients. The data analysis focused on how ALI, as a combined measure of nutritional status and systemic inflammation, correlates with all-cause and CVD mortality. To assess these relationships, we utilized Kaplan–Meier survival curves, Cox proportional hazards regression models, and restricted cubic splines to investigate both linear and non-linear associations between ALI and mortality. We present the findings from these analyses in the following subsections, beginning with baseline characteristics and progressing through specific outcomes related to COPD severity, lung function, chronic pulmonary symptoms, and survival analysis.

### Baseline characteristics

The baseline characteristics of the study participants are summarized in Table 1. A total of 478,800 participants were included, with an average age of 46.99 years (±16.86). The gender distribution showed 52% males and 48% females. Significant differences were observed across groups in several variables, including age, sex, race/ethnicity, family income-to-poverty ratio, education level, smoking status, and chronic disease prevalence such as diabetes, hypertension, cancer, CVD, and COPD.

**Table 1:** Characteristic of study sample.

Characteristic	Overall *N* = 47880^1^	Minimal *N* = 12264^1^	Low *N* = 11323^1^	Intermediate *N* = 11374^1^	High *N* =12929^1^	*p*-value^2^
Age, years	46.99 ± (16.86)	49.85 ± (18.53)	46.82 ± (16.72)	45.93 ± (16.05)	45.34 ± (15.62)	<0.001
Sex						<0.001
Male	24,770 (52%)	6,458 (55%)	5,880 (53%)	5,696 (49%)	6,736 (50%)	
Female	23,110 (48%)	5,806 (45%)	5,433 (47%)	5,678 (51%)	6,193 (50%)	
Race/Ethnicity						<0.001
Mexican American	8,529 (8.2%)	1899 (6.5%)	2,109 (8.0%)	2,276 (9.2%)	2,245 (9.2%)	
Non-Hispanic Black	9,625 (11%)	1,549 (6.3%)	1,597 (7.0%)	2031 (9.1%)	4,448 (20%)	
Non-Hispanic White	21,359 (69%)	6,781 (76%)	5,506 (72%)	4,933 (68%)	4,139 (58%)	
Other Hispanic	3,976 (5.6%)	878 (4.7%)	974 (5.6%)	1,055 (6.3%)	1,069 (5.9%)	
Other Race - Including Multi-Racial	4,391 (6.8%)	1,157 (6.9%)	1,127 (6.8%)	1,079 (7.1%)	1,028 (6.5%)	
Marital						<0.001
Never married	8,246 (17%)	1960 (17%)	1964 (17%)	1907 (17%)	2,415 (18%)	
Married/Living with partner	29,209 (64%)	7,307 (62%)	6,962 (65%)	7,185 (66%)	7,755 (65%)	
Divorced/Widowed/Separated	10,425 (18%)	2,997 (22%)	2,387 (17%)	2,282 (17%)	2,759 (17%)	
Family income poverty ratio	2.99 ± (1.60)	2.96 ± (1.59)	3.04 ± (1.60)	3.06 ± (1.60)	2.92 ± (1.59)	<0.001
Education						<0.001
Less than high school	5,752 (5.9%)	1,423 (5.7%)	1,420 (6.0%)	1,386 (5.9%)	1,523 (6.2%)	
High school or equivalent	18,158 (35%)	4,734 (36%)	4,174 (34%)	4,209 (34%)	5,041 (37%)	
College and above	23,970 (59%)	6,107 (58%)	5,719 (60%)	5,779 (60%)	6,365 (57%)	
Smoking status						<0.001
Former	11,877 (25%)	3,303 (25%)	2,759 (24%)	2,734 (25%)	3,081 (25%)	
Never	26,035 (54%)	6,150 (50%)	6,097 (54%)	6,365 (55%)	7,423 (56%)	
Now	9,968 (21%)	2,811 (25%)	2,457 (22%)	2,275 (20%)	2,425 (19%)	
Drinking status						0.003
Former	7,993 (14%)	2,308 (15%)	1757 (13%)	1808 (13%)	2,120 (14%)	
Mild	9,642 (22%)	2,346 (22%)	2,344 (22%)	2,311 (21%)	2,641 (22%)	
Moderate	16,487 (37%)	4,233 (37%)	3,996 (38%)	3,887 (37%)	4,371 (36%)	
Heavy	6,652 (16%)	1,565 (15%)	1,601 (16%)	1,664 (17%)	1822 (16%)	
Never	7,106 (11%)	1812 (11%)	1,615 (11%)	1704 (12%)	1975 (12%)	
MET scores, min/week	3,394.98 ± (5,074.74)	3,180.68 ± (4,868.35)	3,337.21 ± (5,024.44)	3,463.54 ± (5,153.87)	3,598.55 ± (5,235.94)	<0.001
HEI-2015	50.43 ± (13.25)	50.95 ± (13.44)	50.37 ± (13.20)	50.27 ± (13.28)	50.13 ± (13.07)	0.003
Cancer						<0.001
No	43,589 (91%)	10,679 (87%)	10,323 (91%)	10,524 (92%)	12,063 (93%)	
Yes	4,291 (9.3%)	1,585 (13%)	990 (8.9%)	850 (7.9%)	866 (7.3%)	
CVD						<0.001
No	42,648 (91%)	10,435 (88%)	10,097 (92%)	10,297 (93%)	11,819 (93%)	
Yes	5,232 (8.6%)	1829 (12%)	1,216 (8.2%)	1,077 (7.4%)	1,110 (7.2%)	
DM						<0.001
No	39,697 (87%)	10,249 (87%)	9,537 (89%)	9,402 (88%)	10,509 (86%)	
Yes	8,183 (13%)	2015 (13%)	1776 (11%)	1972 (12%)	2,420 (14%)	
Hypertension						<0.001
No	28,004 (63%)	7,179 (63%)	6,868 (66%)	6,750 (64%)	7,207 (60%)	
Yes	19,876 (37%)	5,085 (37%)	4,445 (34%)	4,624 (36%)	5,722 (40%)	
COPD						<0.001
No	45,920 (96%)	11,510 (94%)	10,881 (96%)	10,979 (97%)	12,550 (97%)	
Yes	1960 (3.9%)	754 (5.8%)	432 (3.6%)	395 (3.2%)	379 (3.1%)	

1 Mean ± error; n (unweighted) (%).

2 An analysis of variance; Pearson’s X^2: Rao & Scott adjustment.

HEI-2015, Healthy Eating Index-2015; MET, Metabolic Equivalent of Task; CVD, cardiovascular disease; DM, diabetes mellitus; COPD, chronic obstructive pulmonary disease.

### ALI and COPD

Table 2 displays the association between ALI and COPD. In the crude model, a lower ALI category was significantly associated with a higher risk of COPD (p for trend = 0.001). After adjusting for age, gender, race, marital status, family income, education, smoking status, alcohol intake, physical activity, HEI-2015 score, and comorbidities (Model 2), this trend was not statistically significant (*p* = 0.086). Compared to the minimal ALI category, the low (OR = 0.81, 95% CI: 0.69–0.95) and intermediate categories (OR = 0.80, 95% CI: 0.69–0.93) were associated with reduced odds of COPD, while the high ALI category showed a weaker but still significant protective effect (OR = 0.85, 95% CI: 0.70–0.98).

**Table 2:** Association between advanced lung cancer inflammation index and COPD.

Outcomes		Crude Model	Model 1	Model 2
		OR (95%CI)	OR (95%CI)	OR (95%CI)
COPD	Continuous	0.95 (0.92, 0.98)	0.98 (0.97, 1.01)	0.99 (0.98, 1.01)
	Categories			
	Minimal	**Ref.**	**Ref.**	**Ref.**
	Low	**0.61 (0.52, 0.70)**	**0.75 (0.65, 0.88)**	**0.81 (0.69, 0.95)**
	Intermediate	**0.54 (0.47, 0.62)**	**0.74 (0.64, 0.86)**	**0.80 (0.69, 0.93)**
	High	**0.52 (0.43, 0.62)**	**0.76 (0.63, 0.91)**	**0.85 (0.70, 0.98)**
	*p* for trend	0.001	0.004	0.086

Crude Model: no covariates were adjusted. Model 1: Adjusted covariates for model 1 included age, gender, race, marital status, family income level, and educational level. Model 2: Adjusted covariates for model 2 included the covariates for model 1 plus smoking status, alcohol intake, physical activity, and HEI-2015 data, diabetes, hypertension, cancer and cardiovascular disease. Bolded results represent statistically significant associations based on a p-value of less than 0.05. COPD, chronic obstructive pulmonary disease; 95%CI, 95% confidence interval.

### ALI and lung function

Supplementary Table S1 examines the relationship between ALI and lung function, measured by FVC and FEV1. In the fully adjusted model (Model 2), no significant association was observed between ALI and either FVC or FEV1. While some improvements in lung function were noted in the intermediate ALI group for FEV1 (*β* = 54.61, 95% CI: 14.34–94.87), the overall trend was not consistent or clinically significant.

### ALI and chronic pulmonary symptoms

Supplementary Table S2 presents the relationship between ALI and chronic pulmonary symptoms, including frequent cough, frequent phlegm, and past-year wheeze. In the fully adjusted model (Model 2), no significant associations were observed between ALI and these symptoms. While lower ALI categories appeared to show reduced odds for frequent cough and phlegm in the crude and partially adjusted models, the associations diminished after full adjustment.

### Kaplan–Meier analysis

**Figure 2:**
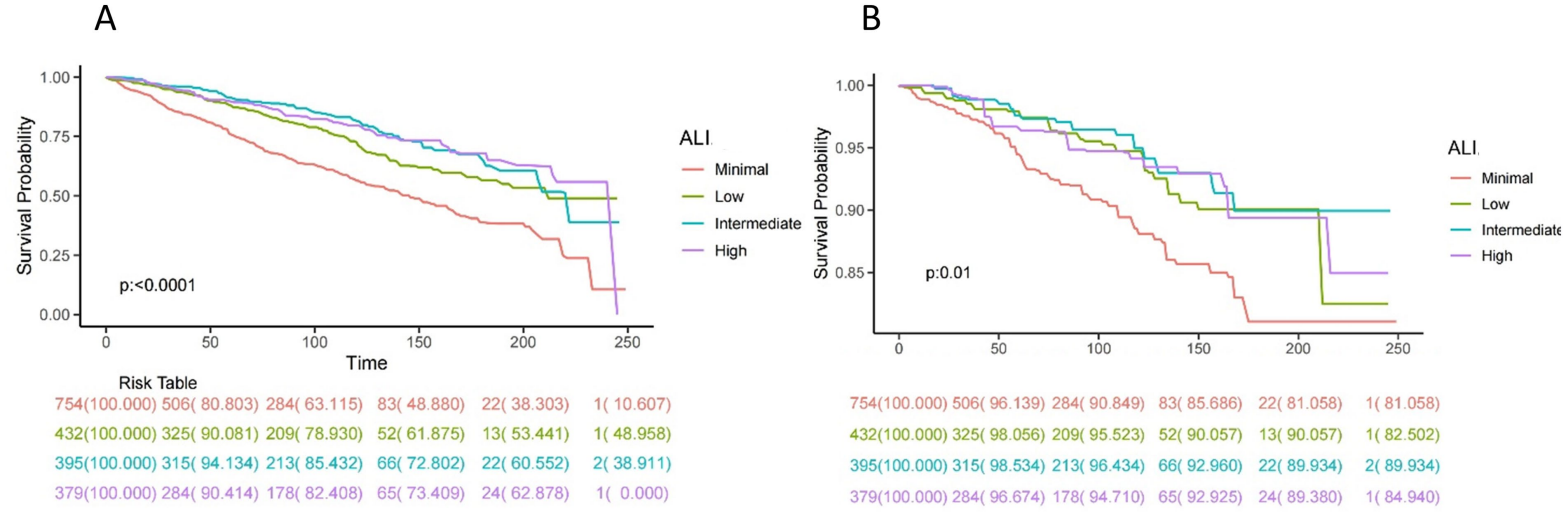
Kaplan–Meier survival curves of ALI impact on long-term all-cause **(A)** and CVD **(B)** mortality in patients with COPD (weighted). ALI, advanced lung cancer inflammation index; CVD, cardiovascular disease.

**Figure 3:**
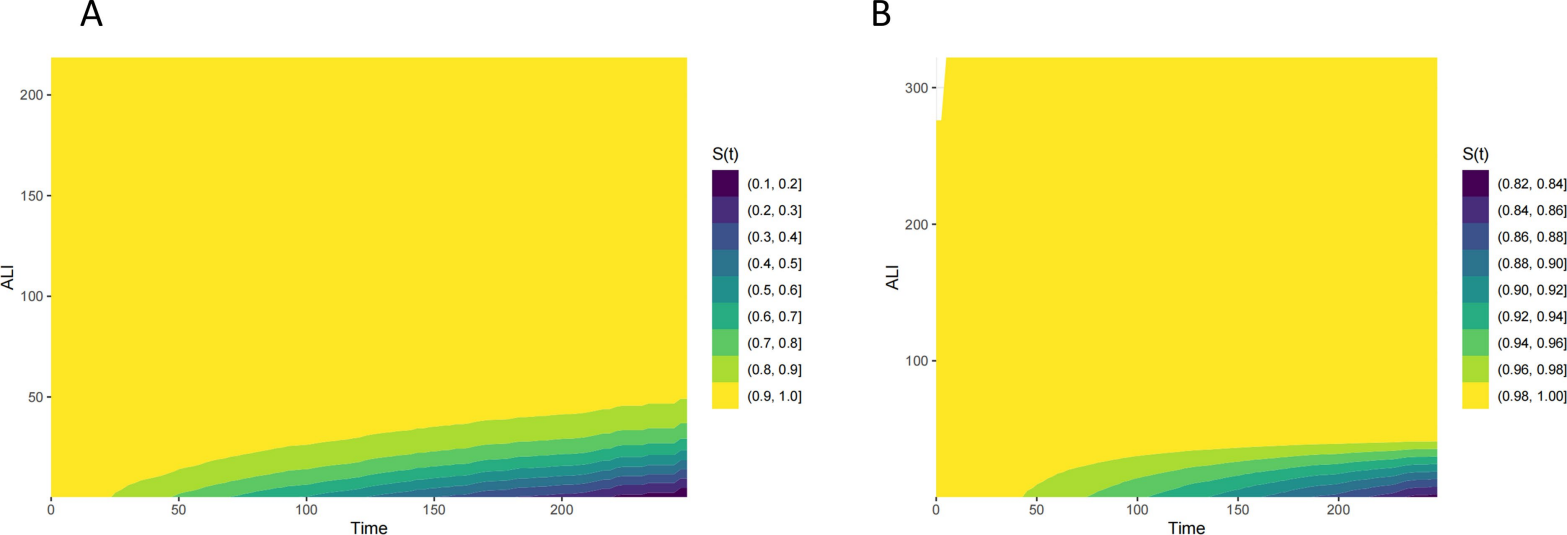
Time-dependent survival probability [S(t)] across ALI levels all-cause **(A)** and CVD **(B)** mortality in patients with COPD.

Figure 2 presents Kaplan–Meier survival curves for all-cause mortality and CVD mortality across different ALI categories. The curves indicate that higher ALI levels are associated with improved survival, with the most pronounced difference observed for all-cause mortality. Patients in the higher ALI categories exhibit better survival probabilities compared to those in the minimal or low ALI categories. Figure 3 illustrates the time-dependent survival probability S(t) across different ALI levels for two groups: all-cause mortality (A) and cardiovascular disease (CVD) mortality (B) in patients with COPD. The heatmap in panel A (All-Cause Mortality) represents the survival probability over time (x-axis) at varying ALI levels (y-axis), with the color gradient reflecting the S(t) value for each combination of time and ALI. A lower S(t) value indicates a lower survival probability, with the color scale ranging from yellow (higher survival probability, closer to 1) to purple (lower survival probability, closer to 0). For all-cause mortality, the survival probability decreases significantly when ALI levels fall below 50. Similarly, panel B (CVD Mortality) shows the time-dependent survival probability for cardiovascular disease mortality, with the color scale again ranging from yellow (higher survival probability) to purple (lower survival probability). Compared to the all-cause mortality panel, the survival probability for CVD mortality is lower at certain ALI levels, with a significant decrease in survival probability when ALI levels fall below 25.

### ALI and mortality

Table 3 examines the relationship between ALI and mortality outcomes, including all-cause and CVD mortality, in patients with COPD. In the fully adjusted model (Model 2), higher ALI categories were significantly associated with lower all-cause mortality. Compared to the minimal ALI category, the low ALI category (HR = 0.68, 95% CI: 0.53–0.87) was associated with a significant reduction in all-cause mortality risk, while the intermediate ALI category (HR = 0.50, 95% CI: 0.38–0.65) exhibited the strongest protective effect. However, in the high ALI category (HR = 0.60, 95% CI: 0.46–0.78), the reduction in mortality risk was slightly less pronounced than in the intermediate group. For CVD mortality, however, no significant association was observed in the fully adjusted model.

**Table 3:** Relationships of advanced lung cancer inflammation index with all-cause and CVD mortality in patients with COPD.

Outcomes		Crude Model	Model 1	Model 2
		HR (95%CI)	HR (95%CI)	HR (95%CI)
All causes	Continuous	0.93 (0.88, 0.98)	0.97 (0.93, 1.02)	0.97 (0.93, 1.02)
	Categories			
	Minimal	Ref.	Ref.	Ref.
	Low	**0.56 (0.43, 0.73)**	**0.69 (0.54, 0.90)**	**0.68 (0.53, 0.87)**
	Intermediate	**0.41 (0.31, 0.54)**	**0.55 (0.43, 0.71)**	**0.50 (0.38, 0.65)**
	High	**0.41 (0.33, 0.52)**	**0.59 (0.45, 0.78)**	**0.60 (0.46, 0.78)**
	*p* for trend	0.001	0.001	0.001
CVD	Continuous	0.92 (0.85, 1.01)	0.98 (0.92, 1.05)	0.98 (0.91, 1.05)
	Categories			
	Minimal	Ref.	Ref.	Ref.
	Low	0.55 (0.32, 0.96)	0.69 (0.39, 1.22)	0.68 (0.39, 1.19)
	Intermediate	0.44 (0.24, 0.81)	0.64 (0.34, 1.18)	0.53 (0.28, 1.00)
	High	0.52 (0.29, 0.95)	0.87 (0.46, 1.66)	0.88 (0.44, 1.76)
	*p* for trend	0.033	0.646	0.593

Crude Model: no covariates were adjusted. Model 1: Adjusted covariates for model 1 included age, gender, race, marital status, family income level, and educational level. Model 2: Adjusted covariates for model 2 included the covariates for model 1 plus smoking status, alcohol intake, physical activity, and HEI-2015 data, diabetes, hypertension, cancer and cardiovascular disease. Bolded results represent statistically significant associations based on a *p*-value of less than 0.05. OR, Odds Ratio; 95%CI, 95% confidence interval.

Tables 4, 5 examine the stratified relationships between ALI and mortality in COPD patients. Table 4 shows that higher ALI levels are consistently associated with a reduced risk of all-cause mortality across most subgroups, including those stratified by age, CVD, diabetes, and cancer, with no significant interactions observed. Table 5, however, reveals that the association between ALI and CVD mortality is less consistent, with significant protective effects observed in some subgroups (e.g., age < 60), but not others. While ALI is a strong predictor of reduced all-cause mortality in COPD patients, its role in reducing CVD mortality is more variable and context-dependent.

**Table 4:** Stratified analyses of the relationships of advanced lung cancer inflammation index with all-cause mortality in patients with COPD.

Characteristics	ALI					
	Minimal	Low	Intermediate	High	*P* for trend	*P* for interaction
Age						0.82
<60	Ref	0.58(0.30, 1.12)	0.35(0.19, 0.64)	0.38(0.20, 0.72)	0.02	
≥60	Ref	0.67(0.52, 0.87)	0.50(0.37, 0.67)	0.52(0.38, 0.70)	0.22	
Gender						0.35
Male	Ref	0.69(0.49, 0.98)	0.50(0.34, 0.72)	0.79(0.51, 1.21)	0.02	
Female	Ref	0.69(0.48, 1.00)	0.50(0.33, 0.77)	0.46(0.31, 0.68)	0.02	
Smoke status						0.96
Never	Ref	0.96(0.45, 2.05)	0.73(0.33, 1.62)	0.46(0.24, 0.90)	0.28	
Former	Ref	0.63(0.45, 0.88)	0.49(0.34, 0.71)	0.58(0.39, 0.86)	0.71	
Now	Ref	0.58(0.38, 0.89)	0.40(0.25, 0.62)	0.72(0.44, 1.18)	0.48	
Hypertension						0.94
No	Ref	0.72(0.45, 1.14)	0.54(0.31, 0.94)	0.53(0.31, 0.91)	0.1	
Yes	Ref	0.64(0.48, 0.85)	0.44(0.32, 0.61)	0.47(0.34, 0.65)	<0.001	
DM						0.66
No	Ref	0.61(0.45, 0.83)	0.49(0.36, 0.69)	0.43(0.29, 0.64)	0.06	
Yes	Ref	0.57(0.35, 0.95)	0.43(0.25, 0.74)	0.57(0.39, 0.83)	0.06	
CVD						0.56
No	Ref	0.56(0.40, 0.79)	0.48(0.33, 0.68)	0.42(0.27, 0.65)	0.06	
Yes	Ref	0.72(0.51, 1.02)	0.44(0.29, 0.69)	0.53(0.34, 0.82)	0.74	
Cancer						0.42
No	Ref	0.69(0.52, 0.93)	0.50(0.37, 0.69)	0.45(0.33, 0.62)	<0.0001	
Yes	Ref	0.47(0.30, 0.74)	0.43(0.25, 0.76)	0.53(0.31, 0.92)	0.3	

The adjusted covariates encompassed age, gender, race, marital status, family income level, educational attainment, smoking status, alcohol consumption, physical activity, HEI-2015 scores, as well as the presence of diabetes, hypertension, cancer, and cardiovascular disease, excluding the stratified variables. CVD, cardiovascular disease; DM, diabetes mellitus; COPD, chronic obstructive pulmonary disease.

**Table 5:** Stratified analyses of the relationships of advanced lung cancer inflammation index with CVD mortality in patients with COPD.

Characteristics	ALI					
	Minimal	Low	Intermediate	High	*P* for trend	*P* for interaction
Age						0.82
<60	Ref	0.58(0.30, 1.12)	0.35(0.19, 0.64)	0.38(0.20, 0.72)	0.02	
≥60	Ref	0.67(0.52, 0.87)	0.50(0.37, 0.67)	0.52(0.38, 0.70)	0.22	
Gender						0.26
Male	Ref	0.60(0.33, 1.10)	0.46(0.21, 0.99)	1.07(0.44, 2.60)	0.05	
Female	Ref	0.75(0.27, 2.07)	0.67(0.23, 1.99)	0.34(0.14, 0.87)	0.45	
Smoke status						0.96
Never	Ref	0.77(0.10, 5.72)	1.00(0.15, 6.82)	0.33(0.11, 1.02)	0.26	
Former	Ref	0.62(0.31, 1.24)	0.62(0.27, 1.46)	0.77(0.34, 1.71)	0.97	
Now	Ref	0.55(0.19, 1.60)	0.31(0.10, 0.95)	0.76(0.20, 2.93)	0.83	
Hypertension						0.99
No	Ref	0.53(0.17, 1.66)	0.29(0.06, 1.41)	0.27(0.11, 0.66)	0.14	
Yes	Ref	0.65(0.36, 1.19)	0.52(0.26, 1.03)	0.82(0.38, 1.77)	0.39	
DM						0.62
No	Ref	0.46(0.22, 0.96)	0.51(0.23, 1.13)	0.70(0.30, 1.64)	0.83	
Yes	Ref	0.62(0.31, 1.25)	0.34(0.12, 0.99)	0.56(0.24, 1.34)	0.11	
CVD						0.69
No	Ref	0.44(0.18, 1.05)	0.54(0.24, 1.20)	0.62(0.22, 1.75)	0.55	
Yes	Ref	0.86(0.44, 1.66)	0.50(0.19, 1.34)	0.87(0.42, 1.79)	0.42	
Cancer						0.11
No	Ref	0.69(0.38, 1.28)	0.57(0.30, 1.07)	0.48(0.25, 0.90)	0.03	
Yes	Ref	0.36(0.11, 1.14)	0.16(0.02, 1.13)	1.02(0.43, 2.41)	0.22	

The adjusted covariates encompassed age, gender, race, marital status, family income level, educational attainment, smoking status, alcohol consumption, physical activity, HEI-2015 scores, as well as the presence of diabetes, hypertension, cancer, and cardiovascular disease, excluding the stratified variables. CVD, cardiovascular disease; DM, diabetes mellitus; COPD, chronic obstructive pulmonary disease.

### Non-linear relationships

**Figure 4:**
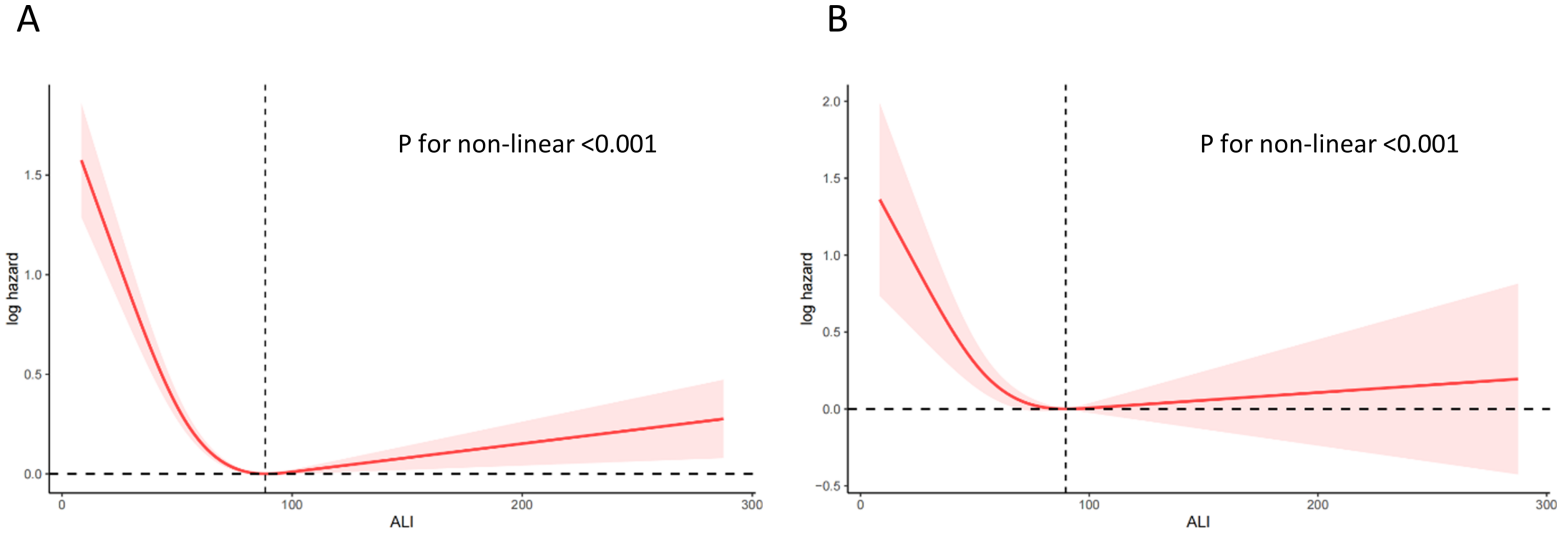
Relationship between ALI and all-cause **(A)** and CVD **(B)** mortality in patients with COPD. The solid and red shadow represent the estimated values and their 95% CIs, respectively. ALI, advanced lung cancer inflammation index; CVD, cardiovascular disease.

**Figure 5:**
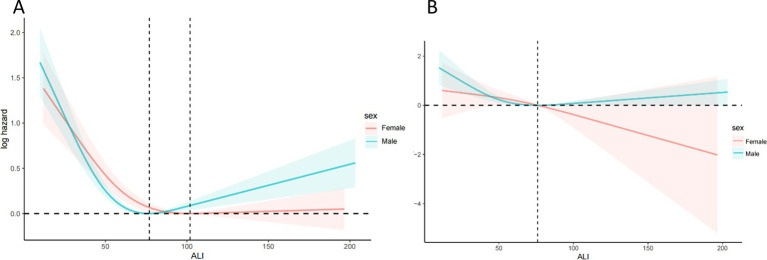
Relationship between ALI and all-cause **(A)** and CVD **(B)** mortality in patients with COPD stratified by sex. The solid and red or blue shadow represent the estimated values and their 95% CIs, respectively. ALI, advanced lung cancer inflammation index; CVD, cardiovascular disease.

Figure 4 showing a clear non-linear association between ALI and both mortality outcomes. The protective effects plateau at higher ALI levels, with a potential increase in mortality risk for very high ALI values. Figure 5 illustrates the sex-stratified relationship between ALI and mortality outcomes in COPD patients. For all-cause mortality, the protective effect of higher ALI levels is consistent across both sexes. For CVD mortality, the relationship is more variable, with clearer protective effects in females than in males.

Tables 6, 7 collectively evaluate the threshold effect of ALI on all-cause and CVD mortality in COPD patients. Table 6 shows that for all-cause mortality, ALI had a significant protective effect when ALI values were below the threshold of 88.32 (HR per 10-unit increment = 0.842, 95% CI: 0.801–0.884, *p* < 0.0001). However, above this threshold, the association became non-significant (HR = 1.012, 95% CI: 0.999–1.024, *p* = 0.062). Similarly, Table 7 highlights that for CVD mortality, ALI was protective below the threshold of 89.73 (HR per 10-unit increment = 0.82, 95% CI: 0.78–0.86, *p* < 0.0001), but the association reversed above the threshold, becoming positively associated with risk (HR = 1.02, 95% CI: 1.00–1.03, *p* = 0.01).

**Table 6:** Threshold effect analysis of advanced lung cancer inflammation index on all-cause mortality in patients with COPD.

	All-cause mortality	
	Per 10 U increment	*P*
<88.32	0.842(0.801, 0.884)	<0.0001
>88.32	1.012(0.999, 1.024)	0.062

Adjusted covariates included age, gender, race, marital status, family income level, educational level, smoking status, alcohol intake, physical activity, and HEI-2015 data, diabetes, hypertension, cancer and cardiovascular disease.

**Table 7:** Threshold effect analysis advanced lung cancer inflammation index on of CVD mortality in patients with COPD.

	CVD mortality	
	Per 10 U increment	*P*
<89.73	0.82(0.78, 0.86)	<0.0001
>89.73	1.02(1.00, 1.03)	0.01

Adjusted covariates included age, gender, race, marital status, family income level, educational level, smoking status, alcohol intake, physical activity, and HEI-2015 data, diabetes, hypertension, cancer and cardiovascular disease. CVD, cardiovascular disease.

### Sensitivity analyses

Table 8 presents a sensitivity analysis that excludes participants who died within 2 years to address potential reverse causation. The results reaffirm that higher ALI levels are significantly associated with reduced all-cause mortality in COPD patients.

**Table 8:** Relationships of advanced lung cancer inflammation index with all-cause and CVD mortality in patients with COPD after excluding participants who died within 2 years.

Outcomes		Crude model	Model 1	Model 2
		OR (95%CI)	OR (95%CI)	OR (95%CI)
All-cause	Continuous	**0.93 (0.88, 0.99)**	0.96 (0.91, 1.01)	0.95 (0.90, 1.00)
	Categories			
	Minimal	Ref.	Ref.	Ref.
	Low	**0.61 (0.46, 0.80)**	**0.68 (0.52, 0.89)**	**0.68(0.53, 0.89)**
	Intermediate	**0.44 (0.33, 0.59)**	**0.53 (0.40, 0.71)**	**0.50(0.36, 0.67)**
	High	**0.44 (0.33, 0.59)**	**0.52 (0.38, 0.71)**	**0.52(0.37, 0.71)**
	*p* for trend	0.001	0.001	0.001
CVD	Continuous	0.92 (0.85, 1.01)	0.99 (0.93, 1.06)	0.98 (0.91, 1.06)
	Categories			
	Minimal	Ref.	Ref.	Ref.
	Low	0.55 (0.32, 0.96)	0.61 (0.32, 1.15)	0.60 (0.32, 1.11)
	Intermediate	0.44 (0.24, 0.81)	0.65 (0.34, 1.27)	0.55 (0.28, 1.09)
	High	0.52 (0.29, 0.95)	0.87 (0.44, 1.72)	0.83 (0.40, 1.76)
	*p* for trend	0.033	0.756	0.626

**Figure 6:**
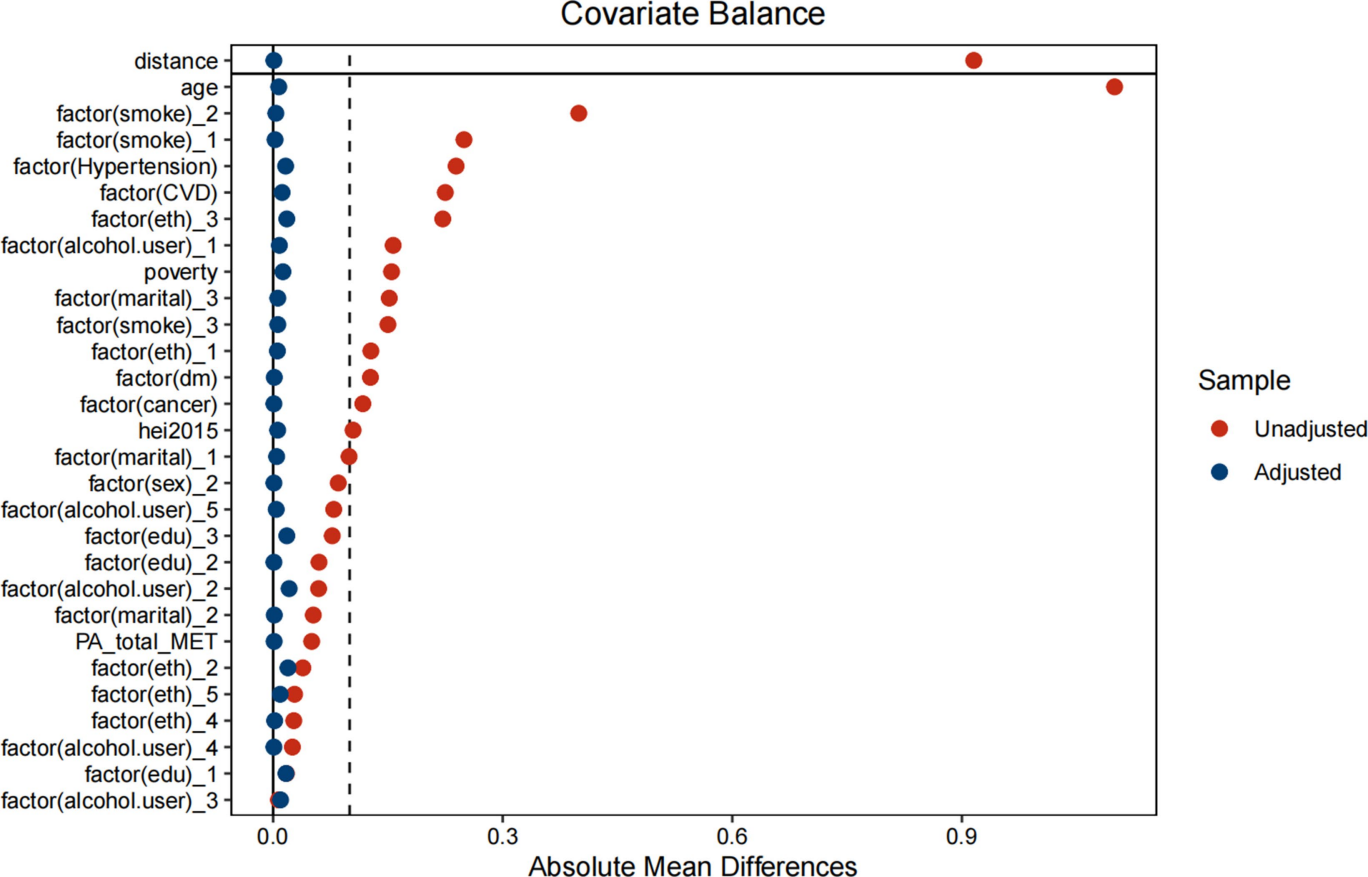
Propensity score matching analysis of the standardized mean difference results for the different variables.

Table 9 indicates significant differences in baseline characteristics between COPD and non-COPD participants before matching, including age, education level, comorbidities (e.g., hypertension, diabetes, and cancer), and smoking status. After PSM, Table 10 and Figure 6 shows that the baseline characteristics were balanced between the two groups, ensuring comparability. Table 11 demonstrates the association between ALI and COPD was similar.

**Table 9:** Basic characteristics of participants before PSM (propensity score matching) analysis.

Characteristic	Overall *N* = 47880^1^	Normal *N* = 45920^1^	COPD *N*= 1960^1^	*p*-value^2^
Age, years	46.99 ± (16.86)	46.43 ± (16.77)	60.66 ± (12.85)	<0.001
Sex				0.133
Male	24,770 (52%)	23,916 (52%)	854 (49%)	
Female	23,110 (48%)	22,004 (48%)	1,106 (51%)	
Age, years				<0.001
Sex	8,529 (8.2%)	8,420 (8.5%)	109 (1.7%)	
Male	9,625 (11%)	9,304 (11%)	321 (6.9%)	
Female	21,359 (69%)	20,068 (68%)	1,291 (83%)	
Age, years	3,976 (5.6%)	3,864 (5.7%)	112 (2.3%)	
Sex	4,391 (6.8%)	4,264 (6.9%)	127 (6.3%)	
Male				<0.001
Female	8,246 (17%)	8,095 (18%)	151 (6.5%)	
Age, years	29,209 (64%)	28,112 (65%)	1,097 (62%)	
Sex	10,425 (18%)	9,713 (18%)	712 (31%)	
Family income poverty ratio	2.99 ± (1.60)	3.00 ± (1.60)	2.74 ± (1.58)	<0.001
Education				<0.001
Less than high school	5,752 (5.9%)	5,484 (5.8%)	268 (8.3%)	
High school or equivalent	18,158 (35%)	17,302 (35%)	856 (42%)	
College and above	23,970 (59%)	23,134 (59%)	836 (50%)	
Cancer				<0.001
No	43,589 (91%)	42,025 (91%)	1,564 (77%)	
Yes	4,291 (9.3%)	3,895 (8.7%)	396 (23%)	
CVD				<0.001
No	42,648 (91%)	41,325 (92%)	1,323 (72%)	
Yes	5,232 (8.6%)	4,595 (7.8%)	637 (28%)	
DM				<0.001
No	39,697 (87%)	38,311 (88%)	1,386 (76%)	
Yes	8,183 (13%)	7,609 (12%)	574 (24%)	
HEI-2015	50.43 ± (13.25)	50.45 ± (13.25)	49.82 ± (13.29)	0.158
Hypertension				<0.001
No	28,004 (63%)	27,307 (64%)	697 (41%)	
Yes	19,876 (37%)	18,613 (36%)	1,263 (59%)	
MET scores, min/week	3,394.98 ± (5,074.74)	3,383.81 ± (5,085.16)	3,669.99 ± (4,804.11)	<0.001
Smoking status				<0.001
Former	11,877 (25%)	10,922 (24%)	955 (47%)	
Never	26,035 (54%)	25,720 (55%)	315 (17%)	
Now	9,968 (21%)	9,278 (21%)	690 (36%)	
Drinking status				<0.001
Former	7,993 (14%)	7,371 (13%)	622 (28%)	
Mild	9,642 (22%)	9,359 (22%)	283 (16%)	
Moderate	16,487 (37%)	15,799 (37%)	688 (36%)	
Heavy	6,652 (16%)	6,427 (16%)	225 (14%)	
Never	7,106 (11%)	6,964 (12%)	142 (6.4%)	

1 Mean ± error; n (unweighted) (%).

2 An analysis of variance; Pearson’s X^2: Rao & Scott adjustment.

HEI-2015, Healthy Eating Index-2015; MET, Metabolic Equivalent of Task; CVD, cardiovascular disease; DM, diabetes mellitus; COPD, chronic obstructive pulmonary disease.

**Table 10:** Basic characteristics of participants after PSM (propensity score matching) analysis.

Characteristic	Overall *N* = 3,795^1^	Normal *N* = 1,930^1^	COPD *N* = 1,865^1^	*p*-value^2^
Characteristic	60.14 ± (14.24)	59.68 ± (15.48)	60.62 ± (12.83)	0.521
Age, years				0.437
Sex	1,692 (48%)	841 (47%)	851 (49%)	
Male	2,218 (52%)	1,114 (53%)	1,104 (51%)	
Female				0.668
Age, years	212 (1.7%)	103 (1.7%)	109 (1.7%)	
Sex	657 (7.0%)	336 (7.1%)	321 (6.9%)	
Male	2,549 (82%)	1,263 (81%)	1,286 (83%)	
Female	193 (2.3%)	81 (2.2%)	112 (2.3%)	
Age, years	299 (6.9%)	172 (7.4%)	127 (6.4%)	
Sex				0.868
Male	277 (6.5%)	126 (6.5%)	151 (6.5%)	
Female	2,266 (63%)	1,171 (63%)	1,095 (63%)	
Age, years	1,367 (31%)	658 (30%)	709 (31%)	
Sex	2.78 ± (1.54)	2.82 ± (1.50)	2.75 ± (1.58)	0.230
Family income poverty ratio				0.895
Education	553 (8.1%)	287 (7.9%)	266 (8.3%)	
Less than high school	1,678 (42%)	824 (42%)	854 (42%)	
High school or equivalent	1,679 (50%)	844 (50%)	835 (50%)	
College and above	62.55 ± (58.94)	63.10 ± (58.12)	61.99 ± (59.79)	0.072
Cancer				0.381
No	3,080 (78%)	1,519 (79%)	1,561 (77%)	
Yes	830 (22%)	436 (21%)	394 (23%)	
CVD				0.248
No	2,673 (73%)	1,350 (74%)	1,323 (72%)	
Yes	1,237 (27%)	605 (26%)	632 (28%)	
DM				0.797
No	2,768 (76%)	1,383 (76%)	1,385 (76%)	
Yes	1,142 (24%)	572 (24%)	570 (24%)	
HEI-2015	49.68 ± (13.00)	49.51 ± (12.72)	49.85 ± (13.28)	0.542
Hypertension				0.842
No	1,403 (41%)	706 (41%)	697 (41%)	
Yes	2,507 (59%)	1,249 (59%)	1,258 (59%)	
MET scores, min/week	3,812.44 ± (5,648.26)	3,949.50 ± (6,356.79)	3,670.64 ± (4,804.14)	0.022
Smoking status				0.646
Former	1939 (48%)	985 (49%)	954 (47%)	
Never	632 (17%)	317 (16%)	315 (17%)	
Now	1,339 (35%)	653 (35%)	686 (36%)	
Drinking status				0.317
Former	1,227 (26%)	609 (25%)	618 (28%)	
Mild	581 (17%)	298 (18%)	283 (16%)	
Moderate	1,389 (37%)	702 (38%)	687 (36%)	
Heavy	435 (14%)	210 (13%)	225 (14%)	
Never	278 (6.0%)	136 (5.6%)	142 (6.4%)	

1 Mean ± error; n (unweighted) (%).

2 An analysis of variance; Pearson’s X^2: Rao & Scott adjustment.

HEI-2015, Healthy Eating Index-2015; MET, Metabolic Equivalent of Task; CVD, cardiovascular disease; DM, diabetes mellitus; COPD, chronic obstructive pulmonary disease.

**Table 11:** Association between advanced lung cancer inflammation index and COPD after PSM (propensity score matching) analysis.

	OR	95%CI	*p*
Continuous	1.00	0.99, 1.01	0.549
Categories	–	–	–
Minimal	Ref.	Ref.	–
Low	0.74	0.59, 0.93	0.01
Intermediate	0.75	0.61, 0.92	0.03
High	0.86	0.68, 1.08	0.16
*p* for trend			0.04

Adjusted MET scores, min/week.

## Discussion

Our study reveals that the ALI is significantly associated with both all-cause mortality and CVD mortality in COPD patients, exhibiting a notable non-linear relationship. Higher ALI levels were protective up to specific thresholds (88.32 for all-cause mortality and 89.73 for CVD mortality); however, beyond these thresholds, the protective effect plateaued and became non-significant for all-cause mortality, while reversing for CVD mortality, indicating increased risk. This non-linear trend underscores the intricate balance between inflammation and nutritional status, suggesting that optimizing ALI within an appropriate range can improve outcomes, whereas excessive levels may reflect underlying pathological changes requiring further investigation.

Chronic inflammation in individuals with COPD is primarily driven by the infiltration of inflammatory cells, including neutrophils, macrophages, and lymphocytes, into the small airways (8). This process leads to the degradation of structural cells such as airway epithelial cells, stromal cells, and parenchymal cells. As COPD progresses, airway inflammation intensifies. The NLR in peripheral blood is a well-established biomarker for quantifying systemic inflammation. Previous studies have explored the relationship between NLR and lung function decline, a key indicator of COPD severity and risk (9). These studies emphasize the clinical relevance of NLR as a biomarker, linking it to impaired lung function, increased COPD risk, and specific DNA methylation patterns. Additionally, research focusing on patients with COPD has demonstrated the prognostic value of hematologic inflammatory markers, such as NLR, in predicting mortality risk (10). Similarly, the platelet-to-lymphocyte ratio (PLR) has been associated with elevated COPD risk (11). These findings highlight the critical role of inflammation in influencing COPD outcomes and align with the conclusions of our study.

Previous research has predominantly focused on individual inflammatory markers to evaluate the prognosis of patients with COPD. However, this approach lacks the comprehensiveness needed to accurately assess the complex relationship between inflammation and mortality risk in COPD. A critical factor contributing to poor prognosis in COPD patients is the high prevalence of malnutrition within this population. A recent meta-analysis of 26 studies found that COPD patients have significantly lower serum albumin levels compared to individuals without COPD (3), with low serum albumin levels being strongly associated with adverse outcomes (12). Furthermore, a large cohort study of 220,000 Chinese men aged 40–79 years, followed over a 15-year period, reported that a 5 kg/m^2^ reduction in Body Mass Index (BMI) was linked to a 31% increase in mortality risk from COPD (13). These findings underscore the importance of simultaneously addressing inflammation and malnutrition in the prognostic evaluation of COPD patients.

The ALI, initially developed as a prognostic marker for survival in non-small cell lung cancer (14), has been applied across various other cancer types, including esophageal, colorectal, pancreatic, and gastric cancers (15–18). Research indicates that cancer survivors with elevated ALI levels and no depressive symptoms face the lowest risks of both all-cause and non-cancer mortality (19). In individuals with type 2 diabetes mellitus (T2DM), elevated ALI levels are strongly associated with reduced risks of all-cause and cardiovascular mortality, particularly among women (20). However, when ALI exceeds a certain threshold, a slight increase in mortality risk may occur. These findings emphasize the importance of weight management and inflammation control in improving the prognosis of individuals with T2DM. Additionally, studies suggest that optimizing ALI levels can significantly reduce cardiovascular mortality in individuals with chronic hypertension, providing valuable insights for managing hypertension-related conditions (21).

The potential biological mechanisms explaining the significantly reduced risk of mortality in individuals with COPD are as follows: Firstly, BMI is a critical indicator of adiposity. Prolonged exposure to cigarette smoke accelerates aging, leading to reduced body weight and premature lung aging (4, 22). This phenomenon may account for the strong association between low BMI and higher COPD-related mortality. A large prospective community cohort study in Japan found that decreased BMI and substantial weight loss were independently associated with an increased risk of COPD mortality (23). Secondly, serum albumin, a protein synthesized by the liver, plays essential roles in the transport and regulation of nutrients, hormones, and medications. Recent studies have shown that reduced serum albumin levels are linked to systemic inflammation activation and an increased risk of malnutrition (24). Additionally, albumin protects tissues from inflammatory damage. Thirdly, neutrophils accumulate in the sputum of stable COPD patients with severe disease, unlike those with mild or moderate COPD. This accumulation is associated with elevated expression of macrophage inflammatory protein-1α (MIP-1α) in bronchial epithelial cells (25). As COPD progresses, increased neutrophil levels are observed in the small airways of COPD patients compared to smokers with normal lung function (26). Lymphocytes also contribute to alveolar destruction in COPD. Specifically, CD8+ T cells produce pro-inflammatory cytokines such as IL-2, interferon-*γ*, and TNFα, which recruit additional inflammatory cells (27, 28). These cells also release perforin and granzyme B, inducing lysis and apoptosis of alveolar epithelial cells and advancing emphysema development (29). In summary, maintaining an appropriate BMI, achieving optimal serum albumin levels, and reducing the NLR can improve ALI scores, thereby supporting a more favorable prognosis in COPD patients.

In this study, we observed that ALI, as a combined measure of inflammation and nutritional status, holds prognostic value in COPD patients. Specifically, lower ALI values are associated with higher risks of COPD, emphasizing the importance of malnutrition and chronic inflammation in this population. Therefore, COPD patients may benefit from interventions aimed at improving nutritional status or reducing inflammation to optimize ALI levels and improve survival outcomes. For patients with very high ALI levels, clinicians should consider the possibility of undiagnosed comorbidities, which may explain the increased mortality risk.

These findings provide new insights for personalized treatment strategies in COPD management. By using ALI as a comprehensive prognostic marker, clinicians can better identify high-risk patients and tailor treatment plans accordingly. For patients with lower ALI levels, nutritional interventions and anti-inflammatory therapies may be prioritized, while those with higher ALI levels may require further assessment of other disease factors. Future research should further validate these thresholds and explore interventions aimed at optimizing ALI, advancing personalized treatment approaches for COPD.

This study has several strengths. First, it utilized data from the NHANES database, which provides a large, nationally representative cohort with comprehensive health and nutrition information, enhancing the generalizability of our findings. Second, by integrating markers of systemic inflammation (neutrophil-to-lymphocyte ratio) and nutritional status (BMI and serum albumin) into the ALI, the study offers a holistic approach to evaluating risk in COPD patients, moving beyond single-biomarker analyses. Third, the use of robust statistical methods, including Kaplan–Meier survival analyses, Cox proportional hazards models, and propensity score matching, strengthens the validity of the results by minimizing potential biases and confounding effects. Finally, the investigation into non-linear relationships between ALI and mortality outcomes provides nuanced insights that can inform clinical risk stratification and personalized interventions.

Despite these strengths, this study also has notable limitations. First, the cross-sectional nature of NHANES data limits the ability to establish causal relationships between ALI and mortality outcomes in COPD patients. Second, ALI relies on serum albumin and BMI, which can be influenced by acute illness or fluid status, potentially confounding its predictive value in chronic conditions like COPD. Third, the study did not include specific inflammatory biomarkers such as C-reactive protein (CRP) or IL-6, which could further refine the understanding of systemic inflammation in this context. Fourth, the thresholds identified for ALI’s protective effects may vary across populations and settings, requiring external validation to confirm their clinical applicability. Lastly, residual confounding cannot be entirely ruled out, despite the comprehensive adjustment for covariates, as certain factors such as genetic predisposition and unmeasured lifestyle variables were not accounted for in the analysis.

## Conclusion

This study underscores the importance of the ALI as a prognostic marker in COPD patients, demonstrating its significant association with all-cause and cardiovascular mortality. The non-linear relationship observed suggests that ALI provides protective effects up to specific thresholds (88.32 for all-cause mortality and 89.73 for CVD mortality), beyond which the association weakens or reverses. These findings highlight the need to address both systemic inflammation and nutritional status in COPD management. Future research should validate these thresholds and explore interventions to optimize ALI, paving the way for more personalized and effective treatment strategies.

## Data Availability

Publicly available datasets were analyzed in this study. This data can be found at: The survey data are publicly available on the internet for data users and researchers throughout the world (www.cdc.gov/nchs/nhanes/).
