# Supplementary material for: Nutritional status and systemic inflammation in COPD: prognostic value of the advanced lung cancer inflammation index
Source: Front Nutr. 2025 Apr 28;12:1550490. doi: 10.3389/fnut.2025.1550490 (PMC12083086; doi:10.3389/fnut.2025.1550490)
Supplement: Supplementary file 1 [file Data_Sheet_1.docx]

Table S1 Association between advanced lung cancer inflammation index and lung function.

| **Outcomes** |  | **Crude Model** | **Model 1** | **Model 2** |
| --- | --- | --- | --- | --- |
|  |  | **Beta (95%CI)** | **Beta (95%CI)** | **Beta (95%CI)** |
| **FVC** | Continuous | -2.55 (-8.02, 2.92) | **-2.33 (-4.42, -0.24)** | **-1.86 (-3.42, -0.30)** |
|  | Categories |  |  |  |
|  | Minimal | Ref. | Ref. | Ref. |
|  | Low | 88.58 (19.57, 157.58) | -12.60 (-58.97, 33.78) | -7.84 (-51.02, 35.35) |
|  | Intermediate | 145.45 (67.73, 223.16) | 2.76 (-39.26, 44.78) | 15.35 (-26.03, 56.73) |
|  | High | 13.26 (-59.73, 86.26) | -30.92 (-76.22, 14.37) | -12.18 (-56.82, 32.47) |
|  | p for trend | 0.409 | 0.266 | 0.846 |
|  |  |  |  |  |
| **FEV1** | Continuous |  |  |  |
|  | Categories | -2.25 (-8.02, 2.92) | 0.08 (-0.45, 0.62) | -0.02 (-0.54, 0.51) |
|  | Minimal | Ref. | Ref. | Ref. |
|  | Low | 119.68 (62.05, 177.31) | 29.99 (-14.12, 74.09) | 25.56 (-18.02, 69.14) |
|  | Intermediate | 191.09 (120.78, 261.40) | 59.55 (17.42, 101.67) | **54.61 (14.34, 94.87)** |
|  | High | 101.62 (42.97, 160.27) | 43.49 (4.33, 82.66) | 38.94 (-0.54, 78.42) |
|  | p for trend | 0.001 | 0.014 | 0.027 |

Crude Model: no covariates were adjusted.

Model 1: Adjusted covariates for model 1 included age, gender, race, marital status, family income level, and educational level.

Model 2: Adjusted covariates for model 2 included the covariates for model 1 plus smoking status, alcohol intake, physical activity, and HEI-2015 data, diabetes, hypertension, cancer and cardiovascular disease.

FEV1, forced expiratory volume in one second; FVC, forced vital capacity; OR, Odds Ratio; 95%CI, 95% confidence interval.

Table S2 Association between advanced lung cancer inflammation index and chronic pulmonary symptoms.

| **Outcomes** |  | **Crude Model** | **Model 1** | **Model 2** |
| --- | --- | --- | --- | --- |
|  |  | **OR (95%CI)** | **OR (95%CI)** | **OR (95%CI)** |
| **Frequent cough** | Continuous | 0.96 (0.94, 0.99) | 0.98 (0.96, 1.00) | 0.99 (0.98, 1.01) |
|  | Categories |  |  |  |
|  | Minimal | Ref. | Ref. | Ref. |
|  | Low | 0.72 (0.61, 0.86) | 0.78 (0.66, 0.94) | 0.85 (0.71, 1.01) |
|  | Intermediate | 0.67 (0.55, 0.81) | 0.75 (0.62, 0.92) | 0.84 (0.69, 1.04) |
|  | High | 0.67 (0.55, 0.81) | 0.77 (0.63, 0.94) | 0.90 (0.74, 1.10) |
|  | *p* for trend | 0.001 | 0.01 | 0.33 |
|  |  |  |  |  |
| **Frequent phlegm** | Continuous | 0.98 (0.96, 1.01) | 1.00 (0.98, 1.01) | 1.00 (1.00, 1.00) |
|  | Categories |  |  |  |
|  | Minimal | Ref. | Ref. | Ref. |
|  | Low | 0.76 (0.65, 0.88) | 0.83 (0.72, 0.97) | 0.90 (0.77, 1.05) |
|  | Intermediate | 0.67 (0.56, 0.81) | 0.77 (0.64, 0.92) | 0.85 (0.71, 1.02) |
|  | High | 0.64 (0.54, 0.77) | 0.74 (0.61, 0.89) | 0.85 (0.70, 1.04) |
|  | *p* for trend | 0.001 | 0.002 | 0.09 |
|  |  |  |  |  |
| **Past year wheeze** | Continuous | 1.00 (0.99, 1.00) | 1.00 (0.99, 1.00) | 1.00 (1.00, 1.00) |
|  | Categories |  |  |  |
|  | Minimal | Ref. | Ref. | Ref. |
|  | Low | 0.98 (0.85, 1.11) | 1.02 (0.89, 1.17) | 1.07 (0.93, 1.23) |
|  | Intermediate | 0.96 (0.85, 1.08) | 1.03 (0.91, 1.15) | 1.09 (0.96, 1.24) |
|  | High | 0.92 (0.82, 1.04) | 0.97 (0.86, 1.10) | 1.05 (0.92, 1.19) |
|  | *p* for trend | 0.218 | 0.694 | 0.482 |

Crude Model: no covariates were adjusted.

Model 1: Adjusted covariates for model 1 included age, gender, race, marital status, family income level, and educational level.

Model 2: Adjusted covariates for model 2 included the covariates for model 1 plus smoking status, alcohol intake, physical activity, and HEI-2015 data, diabetes, hypertension, cancer and cardiovascular disease.

OR, Odds Ratio; 95%CI, 95% confidence interval.
